# Environmental Drivers and Seasonal Dynamics of Spontaneous Plant Communities on Urban Walls: A Case Study in Nanjing, China

**DOI:** 10.3390/plants15040541

**Published:** 2026-02-09

**Authors:** Wenxin Yu, Kaidi Wang, Yunfeng Yang, Sha Li, Yao Xiong

**Affiliations:** 1 College of Landscape Architecture, Nanjing Forestry University, No. 159 Longpan Road, Xuanwu District, Nanjing 210037, China; yuwenxin@njfu.edu.cn (W.Y.); wangkaidi@njfu.edu.cn (K.W.); 2 School of Architecture and Design, China University of Mining and Technology, No. 1 Daxue Road, Tongshan District, Xuzhou 221000, China; 5979@cumt.edu.cn; 3 College of Art and Design, Nanjing Forestry University, No. 159 Longpan Road, Xuanwu District, Nanjing 210037, China; emmabear@njfu.edu.cn

**Keywords:** urban ecology, spontaneous vegetation, plant community composition, environmental factors, seasonal variation, nature-based solutions

## Abstract

As urbanization increasingly compresses ecological spaces, traditional urban greening faces dual challenges of high maintenance costs and diminished ecological functions. Within this context, urban walls—characterized by their widespread distribution, diverse microhabitats, and relatively low levels of human intervention—are gaining recognition as valuable components of urban green infrastructure. Spontaneous wall vegetation, with its strong local adaptability and ecological functions, aligns well with emerging concepts of low-intervention, nature-based urban restoration. This study investigates the composition and environmental drivers of spontaneous wall plant communities across 321 plots on 100 urban walls in central Nanjing, China. Standardized vegetation surveys recorded species composition, cover, and wall-related environmental variables. Variance partitioning, canonical correspondence analysis, and multiple linear regression were applied to elucidate the relationships between plant diversity patterns and environmental factors. Results revealed high species diversity on urban walls, with 163 vascular plant species across 125 genera and 60 families. Retaining walls and spring plots exhibited more complex community structures. Environmental factors collectively explained 58.1% of the variation in plant communities, with wall inherent attributes contributing 23.1%. Diversity indices indicated a moderate level of richness and evenness, with an average Shannon index of 1.3 (0.6–2.5), Simpson index of 0.6 (0.02–0.9), and Patrick index of 1.9 (0.3–3.8). Microstructural attributes such as joint degradation and surface roughness facilitated colonization, highlighting the critical role of microhabitat heterogeneity in community assembly. As one of the first systematic studies on spontaneous vegetation of urban vertical structures in the Yangtze River Delta, this research provides foundational data on urban wall biodiversity and offers valuable insights for integrating native species into green infrastructure planning.

## 1. Introduction

With the accelerating pace of urbanization, cities around the world are facing unprecedented ecological challenges. The United Nations projects that by 2050, approximately two-thirds of the global population will reside in urban areas [1]. Under limited land availability, urban expansion inevitably leads to landscape fragmentation [2], loss of natural habitats [3], and a decline in ecological functions [4]. Urban green spaces are increasingly fragmented by residential, commercial, and industrial development, weakening ecosystem integrity and reducing ecological functions [4]. Concepts such as Urban Ecology, Nature-based Solutions, and Urban Rewilding advocate for reduced human interference and enhanced self-regulation of natural processes in underutilized urban areas [5,6,7]. Within these frameworks, vertical habitats such as walls provide unique opportunities for spontaneous vegetation, which can contribute to biodiversity, microclimate regulation, and urban ecological resilience, despite occupying limited space [8,9].

Within informal green spaces, spontaneous vegetation plays a vital ecological role. These species germinate, grow, and complete their life cycles without intentional human cultivation, and are commonly observed in street corners, vacant lots, rooftops, and wall surfaces, particularly in urban fringe areas [10]. Such vegetation typically demonstrates strong adaptability to urban environmental stresses, including nutrient-poor substrates, alkaline soils, and water scarcity, which enables self-maintenance and spontaneous community assembly. This not only reduces the costs associated with urban greening management [11], but also provides key ecological functions, such as offering habitats for pollinators [12] and birds [13], mitigating roadside pollution [14], and enhancing landscape esthetics. Therefore, rather than focusing solely on the restoration of idealized ecosystems, recognizing, conserving, and managing spontaneous urban vegetation has emerged as a vital component of sustainable urban greening strategies.

Although walls are not natural ecosystems, they serve as unique artificial microhabitats within urban environments. Vegetation commonly establishes in wall cracks and surface microstructures, resulting in discontinuous and highly heterogeneous spatial distribution patterns [15]. Wall material, physicochemical properties, weathering status, pollution levels, and maintenance disturbances collectively influence the survival environment for wall vegetation [16]. Most spontaneous wall vegetation species are locally common with limited abundance, but may also include invasive taxa, ornamental relics, or even endangered species [15,17], thereby conferring both ecological conservation value and landscape potential to wall habitats. Studies have shown that many spontaneous plants, particularly those originating from lithophytic (rock-dwelling) habitats, naturally colonize urban walls and form relatively stable plant communities [18]. Research conducted in various European and Asian cities has documented over one hundred plant species and abundant arthropods inhabiting different types of stone walls, highlighting the significant role these structures play in supporting urban biodiversity [19,20]. Nevertheless, modern urban wall materials have transitioned from traditional stone blocks to concrete, bricks, cement, or tiles, thereby altering structural properties, weathering processes, and microenvironmental regulation, which may influence plant community composition and distribution [21]. Consequently, a systematic investigation of contemporary urban wall plant communities and their environmental responses is warranted.

Nanjing, a city where historical Ming Dynasty city walls coexist with modern architectural walls in the central urban area, presents a unique setting for studying wall vegetation. Its complex terrain and diverse spatial configurations provide abundant vertical substrates for the growth and diversity of spontaneous wall vegetation. Previous studies on spontaneous vegetation have mainly focused on ground ecological spaces such as parks [22], abandoned lands [23] and streets [24], but have given limited attention to the flexible vertical spaces within the city’s three-dimensional structure. Although a study has targeted crevice plants on the Ming city walls [25], systematic studies on the ecological patterns of spontaneous plants on contemporary brick-concrete and cement walls in Nanjing remain scarce.

This study selected 100 urban walls in central Nanjing as research sites to document the species composition and spatial distribution of herbaceous spontaneous plants and the presence of woody seedlings. Environmental variables were recorded at both the wall and microhabitat scales. Based on this, the study addresses the following main research questions:

(i) How do intrinsic wall attributes, external wall features, joint characteristics, and management practices influence the composition and diversity of spontaneous wall vegetation in urban environments?
(ii) How does seasonal variation (spring vs. autumn) affect these relationships?

We hypothesize that wall structural properties such as material, height, and joint density will strongly regulate species diversity and composition, while seasonal conditions will modulate these effects. In particular, retaining walls and walls with higher microhabitat heterogeneity are expected to support greater diversity, and environmental drivers may differ between spring and autumn due to changes in moisture, temperature, and light availability. These questions are addressed using plot-based surveys combined with statistical analyses, including Canonical Correspondence Analysis and multiple linear regression.

## 2. Results

### 2.1. Basic Characteristics of Spontaneous Vegetation

#### 2.1.1. Species Composition and Ecological Traits of Wall-Dwelling Spontaneous Plants

A total of 163 vascular plant species were recorded, belonging to 125 genera and 60 families. The most species-rich families were Asteraceae, Brassicaceae, Poaceae, and Lamiaceae, contributing 10.4%, 4.9%, 4.2%, and 4.2% of the total flora, respectively. These families represent the dominant floristic components in the surveyed wall habitats. The ten most frequently recorded species were *Broussonetia papyrifera* (11.7%), *Pteris multifida* (11.5%), *Parthenocissus tricuspidata* (7.3%), *Youngia japonica* (5.4%), *Pleuropterus multiflorus* (4.9%), *Acalypha australis* (3.2%), *Solidago canadensis* (2.8%), *Veronica hederifolia* (2.7%), *Causonis japonica* (2.6%), and *Corydalis edulis* (2.3%). These species formed the core assemblage of spontaneous greening in Nanjing’s vertical spaces.

**Figure 1:**
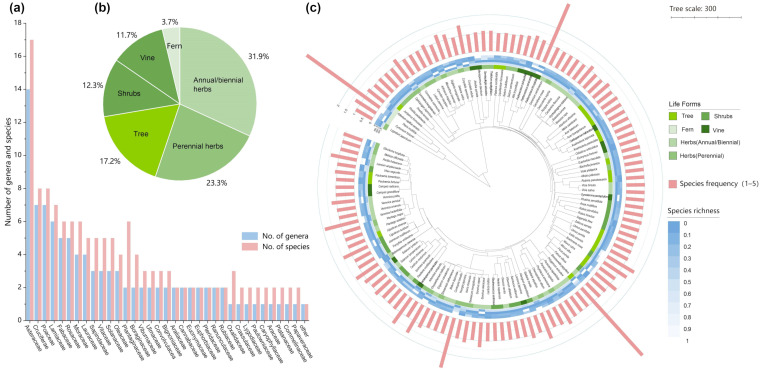
Basic characteristics of spontaneous wall vegetation in the study area. (**a**) Number of families, genera, and species of wall-colonizing spontaneous vegetation. (**b**) Proportional distribution of plant life forms. (**c**) Phylogenetic tree showing relationships among species based on life form, species frequency, and species richness.

Species richness ranged from 1 to 17 species per quadrat, with an average of 4 species, and from 3 to 38 species per wall, with an average of 14 species, indicating substantial variation in local plant diversity (Figure 1a). Herbaceous species dominated the flora (Figure 1b): annuals and perennials together accounted for 55.2%, followed by woody plants (29.4%), vines (11.7%), and ferns (3.7%). Wall structures provided climbing supports for vines and sheltered niches for ferns, suggesting that vertical surfaces may offer ecological buffering and refuge for specific life forms.

#### 2.1.2. Phylogenetic Structure and Dominance Patterns of Wall Dwelling Plants

To examine the phylogenetic composition and functional traits of wall vegetation, we constructed a phylogenetic tree using the complete species list compiled from all species recorded in the present survey and integrated three data layers: life form, species richness, and occurrence frequency. The tree is displayed as a circular plot (Figure 1c). The inner ring denotes life form categories, illustrating how different functional groups are distributed against an evolutionary background. The middle ring shows abundance, expressed as the number of quadrats in which each species occurred within the various wall types. The outer ring represents occurrence frequency across all sampling points. The phylogeny reveals that herbaceous species are widely scattered throughout the tree, indicating broad ecological adaptability across diverse evolutionary lineages. In contrast, vines, shrubs, and trees are more phylogenetically clustered, suggesting stronger constraints imposed by shared ancestry. Core species—such as *Pteris multifida*, *Parthenocissus tricuspidata*, and *Youngia japonica*—rank highest in abundance and occupy central positions in the phylogeny, underscoring their dominance within the urban wall ecosystem. Species with lower frequencies are likely recent colonists or occasional residents; their ecological roles require further assessment in relation to habitat succession and human disturbance.

### 2.2. Environmental Drivers of Wall Plant Community Composition

#### 2.2.1. Variation Partitioning Analysis of Environmental Factors and Plant Composition

The mean species cover of all quadrats within each wall was calculated as the vegetation attribute for that wall. Cover for each species was estimated using typical canopy diameter values obtained from published floras and plant trait databases, providing a standardized approximation of species cover for comparative analyses across walls [26]. Based on plant data from walls, a site × species cover matrix was constructed to analyze vegetation–habitat relationships. The habitat factor matrix comprised four major groups of wall-related environmental factors, totaling 17 variables. Prior to analysis, all data were standardized using z-scores.

**Figure 2:**
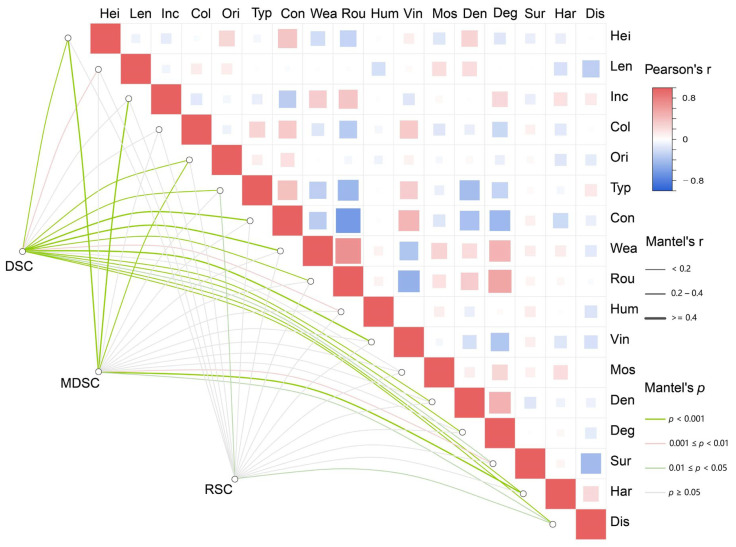
Correlation heatmap of environmental factors. The matrix displays Pearson correlation coefficients between habitat variables. Abbreviations are defined as follows: Hei = wall height; Len = wall length; Inc = wall inclination; Col = wall color; Ori = wall orientation; Typ = wall type; Con = wall construction material; Wea = weathering; Rou = surface roughness; Hum = humidity level; Vin = vine coverage; Mos = moss/lichen coverage; Den = density of joints; Deg = degradation of joints; Sur = surrounding habitats; Har = wall hardening; Dis = human disturbance. The color of each square represents the direction (blue = positive, red = negative) and intensity (darker = stronger) of correlations. Overlaid lines indicate Mantel test results between environmental factors and plant groups: green lines represent significant positive correlations, red lines indicate significant negative correlations, and gray lines denote non-significant relationships.

To better understand the relationship between environmental factors and plant composition, cluster analysis was first conducted to classify quadrats into three plant groups: Dominant Species Cluster (DSC), Moderately Distributed Species Cluster (MDSC), and Rare Species Cluster (RSC), representing species with strong, moderate, and high dependence on wall environments, respectively. Mantel tests were then used to assess correlations between these plant groups and environmental variables, while Pearson correlation was applied to detect multicollinearity among environmental factors, helping to identify suitable variables for subsequent analysis. As shown in Figure 2, wall surface roughness was significantly positively correlated with weathering and joint degradation, but negatively correlated with wall construction material—consistent with the coding of construction material from rough to smooth surfaces. This suggests that finely constructed walls typically have smoother surfaces and fewer crevices, limiting plant attachment. In the Mantel tests, DSC showed the strongest correlations with environmental factors, indicating higher environmental sensitivity, whereas RSC had mostly non-significant associations. MDSC showed intermediate responses, revealing clear differences in how plant groups respond to wall habitat conditions.

#### 2.2.2. Seasonal Variation in Wall Plant Composition Revealed by Canonical Correspondence Analysis

**Figure 3:**
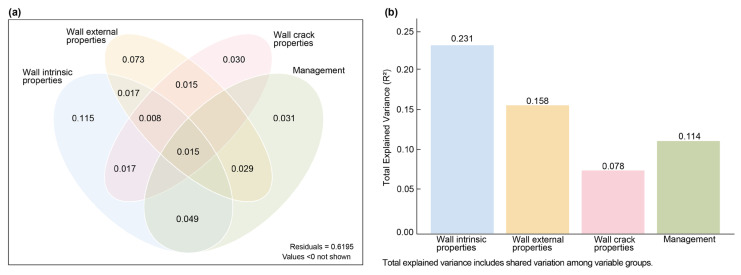
Variance partitioning of species richness explained by four groups of environmental variables: (**a**) Venn diagram showing the proportion of variation independently and jointly explained by wall-inherent, wall-external, joint, and management variables; (**b**) bar plot illustrating the total explained variance (R^2^) by each variable group, including both unique and shared components. All results are based on redundancy analysis (RDA) and were statistically significant (permutation test, F = 12.559, *p* = 0.001, 999 permutations).

Variance partitioning was conducted using the varpart function from the vegan package in R 4.4.3 to quantify the contributions of four groups of environmental factors to the spatial variation in spontaneous wall plant composition. The results indicate that these four groups collectively explain 58.1% of the variation in plant community composition and spatial heterogeneity, leaving 41.9% unexplained (Figure 3). Among the groups, wall inherent attributes account for the largest proportion of explained variation (23.1%), followed by wall external attributes, management measures, and joint attributes. These findings highlight the pivotal role of the structural properties of walls in regulating plant distribution and diversity, especially through their interactions with external wall features, which significantly influence community assembly and succession processes. Additionally, surrounding management practices partially shape the growth environment, thereby affecting species diversity and spatial distribution patterns.

DCA indicated a first axis gradient length of 3.23 (Table 1), exceeding the commonly accepted threshold of 3 [27], suggesting a unimodal species–environment relationship. Therefore, CCA was employed to explore the influence of habitat variables on species distribution patterns. Forward selection and permutation tests were used to identify significant predictors in annual, spring, and autumn datasets. Several variables (e.g., wall structure, vine coverage, wall roughness, and wall type) exhibited strong correlations with the first ordination axis, indicating their key roles in shaping plant communities. Categorical variables were dummy-coded (e.g., sunny aspects = 0, others = 1) to ensure model interpretability and avoid spurious ordinal effects.

**Figure 4:**
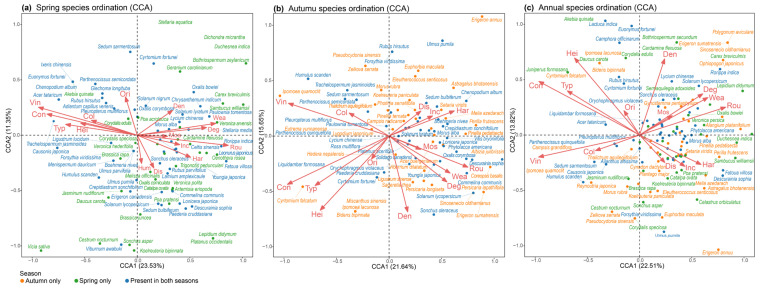
Seasonal variation in CCA ordinations of spontaneous wall vegetation. (**a**) CCA for spring samples; (**b**) CCA for autumn samples; (**c**) CCA for all-year samples. Environmental variables are represented by red arrows. Plant species occurring in both seasons are shown in blue, those specific to spring in green, and those specific to autumn in orange. Variable abbreviations are defined in Table 2.

Species distribution patterns revealed distinct seasonal variations (Figure 4). In spring, communities were dominated by short-lived annuals such as *Cardamine flexuosa* and *Veronica hederifolia*, which clustered along vectors representing wall roughness and humidity. These conditions, typical of older and less-maintained walls, favor low to moderate moss development and moisture retention, providing suitable microhabitats for early spring specialists. In contrast, autumn species exhibited tighter clustering, suggesting more consistent responses to habitat constraints such as dryness and limited resources. Species like *Setaria viridis* and *Cyrtomium fortunei* were closely associated with vectors for wall structure and hardened wall bases, indicating a preference for structurally stable environments. These bases likely provide shade and localized moisture buffering during periods of reduced rainfall. Year-round ordination showed certain species, including *Rubus hirsutus* and *Parthenocissus tricuspidata*, occupying intermediate positions between spring and autumn, suggesting broader ecological niches. As climbing species, they utilize vertical surfaces, crevices, and shading, adapting well to spatial heterogeneity in light, water, and temperature. Their growth forms confer ecological advantages, especially in vertical greening applications.

**Table 2:** Classification and description of environmental variables used in the analysis.

Factor Groups	Factor	Indicator	Range
Wall intrinsic properties	Height	Hei	1.7–12 m
	Length	Len	6–251 m
	Inclination	Inc	1–3
	Color	Col	G = Gray; W = White; Y = Yellow; R = Red
	Orientation	Ori	S; SW; SE; W; E; NW; NE; N
	Type	Typ	R = Retaining wall; S = Standalone wall; B = Building wall; C = City wall
	Construction material	Con	ST = Stone; BR = Brick; CO = Concrete
	Weathering	Wea	1–5
	Roughness	Rou	1–5
Wall external properties	Humidity level	Hum	1–5
	Vine coverage	Vin	1–5
	Moss/lichen coverage	Mos	1–5
Wall crack properties	Density of joints	Den	1–5
	Degradation of joints	Deg	1–5
Management	Surrounding habitats	Sur	1–5
	Hardening	Har	1–5
	Disturbance	Dis	1–5

Overall, wall plant communities demonstrated strong seasonal turnover, shaped by shifting microenvironmental conditions. Climbing plants-maintained presence across seasons, emphasizing their adaptability and functional importance in urban wall habitats.

### 2.3. Diversity Dynamics and Driving Mechanisms

#### 2.3.1. Seasonal Shifts in Wall Plant Diversity

To avoid overfitting in CCA models, four categorical variables—wall type, color, construction material, and aspect—were converted into binary (dummy) variables. Wall type, reflecting physical structure and exhibiting strong explanatory power on CCA axis 1, was selected as the primary grouping variable to examine seasonal diversity changes. Quadrat data were classified by season (spring, autumn) and wall type (retaining, non-retaining) into four groups: spring retaining walls (SRW), spring non-retaining walls (SNRW), autumn retaining walls (ARW), and autumn non-retaining walls (ANRW). Multiple diversity indices were compared across these groups.

**Figure 5:**
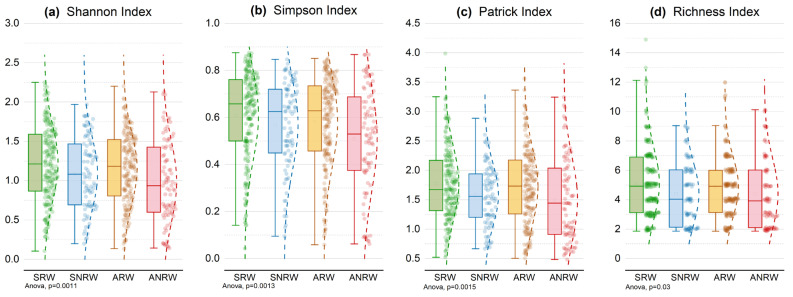
Plant diversity indices across seasons and wall types. (**a**) Shannon index; (**b**) Simpson index; (**c**) Patrick index; (**d**) Species richness. Quadrats were categorized into four groups based on season and wall type: SRW (spring retaining walls), SNRW (spring non-retaining walls), ARW (autumn retaining walls), and ANRW (autumn non-retaining walls). Group differences were tested using one-way ANOVA; significance levels are indicated in the figure.

As shown in Figure 5, SRW exhibited the highest values for all diversity indices—Shannon, Simpson, Patrick, and species richness. The mean Shannon index reached about 2.0, significantly higher than SNRW and both autumn groups. Simpson values indicated greater evenness in retaining wall communities, while ANRW showed the lowest evenness. Patrick and richness indices confirmed that SRW harbored nearly twice the species number compared to ANRW.

Ecologically, spring provides abundant rainfall and mild temperatures that promote germination, rapid growth, and coexistence, especially of short-lived early-spring annuals that quickly complete their life cycles. Structurally, retaining walls are thick, stable, and fissured, retaining moisture and offering three-dimensional microsites ideal for spontaneous plants to root and expand, supporting a more complete vertical niche system. In contrast, autumn is drier and resources are scarcer, favoring drought-tolerant or late-reproducing species. Non-retaining walls, being more exposed and structurally loose, limit colonization and support lower diversity. Even in autumn, retaining walls maintain relatively high richness and evenness, highlighting their ecological buffering capacity. Overall, wall plant diversity varies markedly across both temporal and spatial gradients. Retaining walls in spring—due to their structural stability and favorable microclimate—exhibit the highest diversity and should be prioritized in vertical greening strategies.

#### 2.3.2. Regression Analysis of Environmental Drivers of Wall Vegetation Diversity

**Figure 6:**
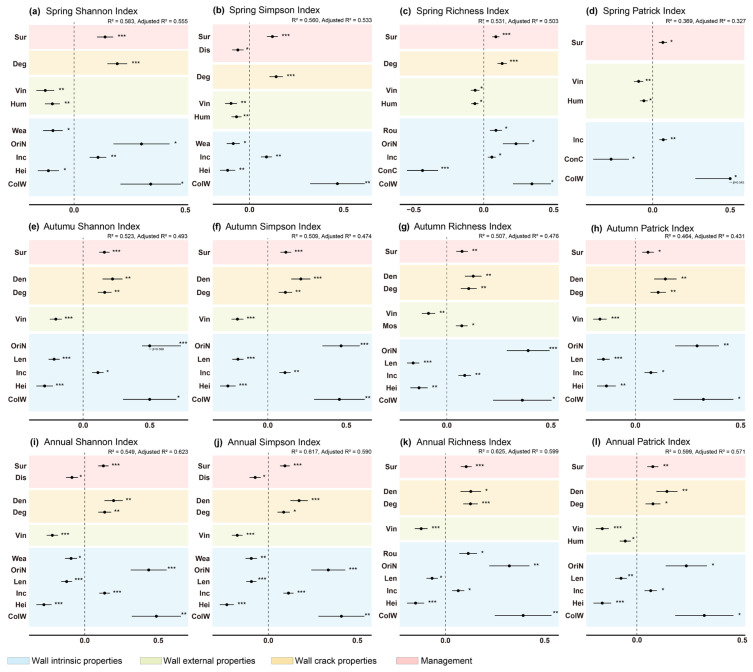
Multiple linear regression analysis of environmental variables and plant diversity across seasonal wall samples. Panels (**a**–**d**) show Shannon, Simpson, richness, and Patrick indices for spring; (**e**–**h**) for autumn; and (**i**–**l**) for the whole year. Only significant predictors (*p* < 0.05) are displayed. Significance levels are indicated as follows: *p* < 0.05 (*), *p* < 0.01 (**), *p* < 0.001 (***). Note: OriN = wall orientation north; ConC = wall construction material concrete; ColW = wall color white. All categorical variables were coded as dummy variables based on their ecological relevance. Abbreviations for other environmental variables are listed in Table 2.

Multiple linear regression models were used to explore the effects of 17 wall-related environmental variables on diversity indices across three temporal scales (Figure 6). To fully capture nuanced differences within categorical factors, categorical variables such as wall type, color, construction material, and orientation were decomposed into multi-level dummy variables. The results show that the determination coefficients (R^2^) of most seasonal models exceed 0.50, indicating that environmental variables collectively explain over half of the variation in diversity key coefficients remained consistent and stable across models. representative models were subjected to residual normality tests, heteroscedasticity tests, multicollinearity diagnostics, and influential point analyses. The results indicate that residuals largely conform to normality, no significant heteroscedasticity was detected, multicollinearity was low, and no influential outliers were found, supporting the robustness and explanatory strength of the models. Although spatial autocorrelation among walls was not explicitly tested due to the relatively large and heterogeneous sampling design, this remains a potential limitation and should be considered when interpreting the results.

Several core predictors exhibited consistent and significant effects throughout the year. Vine coverage on walls consistently reduced all diversity indices across spring, autumn, and full-year datasets. Wall height also remained a stable negative predictor, while north-facing walls and joint degradation showed consistent positive effects. Distinct seasonal differences were also apparent. In spring, wall moisture and the degree of surrounding management emerged as important variables. In autumn, wall length negatively affected diversity, whereas joint density and moss coverage were positive predictors. When analyzing the full-year dataset, wall surface roughness was positively associated with diversity, whereas wall weathering and human interference acted as negative factors.

Overall, joint degradation, wall height, and vine coverage consistently formed the core framework shaping plant diversity across seasons.

## 3. Discussion

### 3.1. Distribution Patterns and Diversity Characteristics of Urban Wall Vegetation

This study surveyed 100 urban walls in the main urban area of Nanjing, establishing 321 standard sampling plots and recording 163 vascular plant species across 125 genera and 60 families. The results reveal a unique plant assemblage characteristic of vertical urban green spaces. Diversity indices showed an average Shannon diversity of 1.3 (range 0.6–2.5), Simpson index of 0.6 (range 0.02–0.9), and Patrick index of 1.9 (range 0.3–3.8), indicating that despite the constraints of the wall environment, plant communities maintain a moderate level of richness and evenness. Compared to other urban green spaces in Nanjing, wall vegetation exhibits specific compositional features. Urban parks [28] recorded 284 species—substantially more than the walls—but despite the lower total species count, wall vegetation still showed considerable taxonomic diversity, spanning 60 families. This indicates that even in spatially restricted vertical habitats, a wide variety of plant lineages can be supported. Across different urban habitat types, Asteraceae and Poaceae consistently emerged as dominant families, reflecting their strong ecological adaptability and flexible reproductive strategies. Their consistent presence underscores the niche advantage of these spontaneous species within vertical green spaces, suggesting potential similarities with species found in horizontal habitats. In terms of life form composition, herbaceous plants accounted for the majority of the wall vegetation surveyed. However, walls hosted a higher proportion of annual and biennial herbs (31.9%) than perennials (23.3%), suggesting a “fast-growth–fast-turnover” community structure in wall vegetation, where “turnover” refers qualitatively to rapid replacement of individuals and species due to short life cycles and environmental filtering. This pattern is likely shaped by the combined effects of spatial limitation, water stress, and human disturbance [29], highlighting the dominant role of environmental filtering in community assembly processes.

Further comparisons show that Nanjing’s urban wall flora shares notable similarities with that of Chongqing (239 species) in terms of species composition and life-form structure [21]. Although total species counts differ, this mainly reflects differences in sampling and urban structure: Chongqing spans multiple districts with varied topography, while Nanjing covers the flat central area with widely distributed sampling points. The resulting species counts were 239 and 163, respectively. Local richness per quadrat was higher in Chongqing, but per-wall richness was similar, suggesting Nanjing walls support comparable species composition despite lower plot-level diversity. This pattern likely reflects the influence of city-scale landscape diversity and habitat heterogeneity, with vertical habitat structure and spatial continuity contributing to cumulative species accumulation rather than driving species richness through scale effects. This trend is particularly evident in Nanjing, highlighting the importance of scale effects in vertical urban green spaces [30]. Notably, the ten most frequently recorded species in this study (e.g., *Broussonetia papyrifera*, *Oxalis corniculata*, and *Sonchus oleraceus*) were also commonly found on Chongqing’s city walls, suggesting that these species have strong adaptive traits suited for vertical environments and may serve as structural species in wall plant communities. This pattern may be interpreted as an instance of biotic homogenization [31], indicating that while urban wall habitats display local variability, their plant communities share many species due to broad geographic distribution and wide ecological amplitude. Historic walls had 159 species, 86 overlapping with modern walls (~54%). Modern walls are dominated by herbaceous species, while historic walls have more woody plants (51.6%), likely reflecting longer establishment times and lower disturbance. Modern walls had 26 non-native species (15%), compared to 15 (9.4%) on historic walls. These species tend to occur in disturbed or high-traffic areas, suggesting walls can act as transitional habitats for disturbance-tolerant exotics. Overall, compared with previous studies mainly focused on historic city walls, this research systematically covers various common urban wall types in Nanjing’s core area—including brick-concrete, cement, and retaining walls—providing a comprehensive overview of their plant assemblages. Observed differences among wall types suggest potential influences on community composition, offering preliminary perspectives on the vegetation carrying capacity of urban vertical spaces. It helps fill the knowledge gap resulting from the historic-wall-centric bias in prior research and highlights the ecological significance of urban gap spaces as habitat.

### 3.2. Influence of Environmental Factors on Urban Wall Plant Community Structure and Diversity

To identify the relative influence of different environmental factors on urban wall plant community composition, this study employed variance partitioning analysis, categorizing wall-related variables into four groups: intrinsic wall attributes, external wall attributes, joint characteristics, and management measures. Among these, intrinsic wall attributes exhibited the highest explanatory power for plant differentiation (20.9%), significantly surpassing external attributes, management, and joint features, indicating that the fundamental structural conditions of walls play a dominant role in plant selection and community assembly. This finding aligns with niche construction theory [32], which posits that the physical habitat structure acts as a strong environmental filter for species colonization and is a primary driver shaping plant spatial patterns in urban ecosystems. Compared with a similar study in Chongqing, where variance partitioning explained only 14.4% of compositional variation, the Nanjing results demonstrate markedly higher explanatory power (53.6%), likely due to more diverse wall types and broader seasonal coverage in this study. Despite methodological differences, both studies confirm that intrinsic wall attributes constitute the primary ecological factor across cities, underscoring the widespread ecological significance of wall structural conditions.

CCA further elucidated the specific responses of plant species to environmental variables. The first two axes of the annual model, which combines species recorded in both spring and autumn surveys, explained 37.97% of the total variation, while the spring and autumn models explained 35.57% and 39.74%, respectively, indicating limited seasonal differences in overall explanatory power, but notable seasonal shifts in dominant factors. Seasonal differences influenced the importance of drivers. In spring, higher wall moisture unexpectedly reduced diversity due to dense moss dominance suppressing seedling establishment, while lower-intensity surrounding management promoted diversity by providing spontaneous vegetation, litter, and exposed soil. In autumn, wall length negatively affected diversity because longer walls are more uniform with lower joint density, whereas higher joint density positively influenced diversity. Moss coverage was a positive predictor only in autumn, likely buffering temperature and moisture fluctuations during drier periods. The annual model consistently identified wall structure, vine coverage, and weathering as stable drivers over broader temporal scales. Comparison with results from Chongqing revealed several consistent patterns. Vine coverage emerged as a consistently negative factor across seasons and the annual model. Dominant vine growth often overgrows vertical space, suppressing herbaceous and annual seedling establishment, thereby reducing overall community diversity. This effect was stable in both spring and autumn, highlighting the competitive exclusion imposed by climbing plants under varying seasonal conditions. Human disturbance exhibited moderate explanatory power in both Nanjing and Chongqing, indicating its regulatory role under frequent human activity in built environments. North-facing walls were positively associated with diversity, likely due to receiving less direct sunlight and maintaining more stable microclimatic conditions that favor species coexistence. Joint degradation showed a significant positive effect, particularly in autumn. Increased structural heterogeneity provides more ecological niches and microhabitats for plant colonization, and can enhance capillarity and moisture retention during alternating wet-dry cycles, facilitating seedling survival. Notably, certain significant variables in Nanjing—such as wall roughness in spring and joint degradation in autumn—did not appear in the Chongqing study, representing important new findings. Surface roughness can enhance the bio receptivity of materials [33], facilitating plant attachment and germination. Meanwhile, degraded joints may enhance capillarity and moisture accumulation, buffering plant survival during the alternating wet-dry cycles in autumn. The diversity of wall types in Nanjing—including brick-concrete, cement, and retaining structures—may amplify interactions between wall microstructure and seasonal drivers, revealing a high degree of fine-scale heterogeneity within urban vertical habitats.

This study also found that urban wall plant diversity is jointly influenced by wall structure and season, exhibiting a spatial-temporal differentiation pattern of “higher diversity in spring than autumn, and higher in retaining walls than non-retaining walls.” This pattern was supported statistically across diversity indices and mechanistically validated by multiple linear regression and CCA models. Regression models incorporating 17 environmental variables demonstrated strong explanatory power for wall plant diversity, identifying vine coverage, wall height, wall orientation, and joint structural condition as core regulatory factors. These findings deepen and confirm insights from variance partitioning and CCA analyses. Specifically, vine coverage exerted a significant negative effect, consistent with competitive exclusion theory [34], where dominant climbers suppress resource access for other species. This aligns with CCA results showing vine shading inhibits herbaceous and annual seedling establishment, reducing community diversity. The negative impact of wall height reflects vertical gradient constraints: taller walls are exposed to stronger winds and solar radiation, with more pronounced temperature and humidity fluctuations, creating harsher conditions unfavorable for many species, thus favoring dominance by a few tolerant species and lowering overall diversity. Conversely, north-facing walls positively influenced diversity, likely due to more stable microclimates conducive to species coexistence. Joint degradation showed a significant positive effect, emphasizing the crucial role of microstructure in community formation. Previous studies highlight that successful vascular plant establishment on masonry walls depends heavily on the presence of suitable substrate, moisture availability, and favorable orientation within cracks or joints [35]. Weathering and mortar loss accumulate substrate within joints, creating diverse microhabitats that facilitate herbaceous root penetration and spread [36]. This micro-scale heterogeneity’s ecological significance aligns with the variance partitioning result emphasizing intrinsic wall attributes as primary drivers, reinforcing the importance of physical wall structure in shaping spontaneous plant distribution. Retaining walls consistently supported higher diversity due to richer joint structures, moderate height, diverse orientations, and more weathered or rough surfaces. During spring, when annual plants germinate, these structural advantages interact with seasonal conditions to create a peak in plant diversity, providing both stable colonization space and favorable microclimatic buffering. In addition to these structural attributes, wall construction material also influences plant colonization through its physical and chemical properties. Porous or weathered materials enhance water retention and provide micro-crevices, while chemical composition affects species establishment and succession [37]. These properties collectively determine the bioreceptivity of the wall surface, contributing to the observed higher diversity on retaining walls. This finding resonates with ecological theories that complex habitats better maintain biodiversity [38], and corresponds with previous results showing significantly greater spring diversity on retaining walls. In summary, this study reveals a multi-dimensional driving mechanism by which urban wall environmental factors influence plant community structure and diversity across scales and models. It highlights the critical role of wall microstructure in urban vertical greening ecology, providing essential theoretical and empirical support for future green infrastructure design.

### 3.3. Ecological Adaptation Mechanisms of Wall Plants and Their Potential for Local Greening

The survey of urban walls in Nanjing reveals that wall surfaces have become unique habitats capable of supporting diverse plant communities [39], exhibiting distinct dominance patterns. Wall plants can establish, expand, and renew themselves within highly anthropogenic vertical spaces, and their ecological adaptation mechanisms merit in-depth investigation. Survival in extreme microhabitats on walls depends largely on species’ responsiveness to structural crevices, moisture availability, and light conditions. Seeds typically disperse onto wall microcracks via wind, water flow, or birds [40,41], germinating in weathered fissures, masonry joints, or rough surfaces. The wall’s surface roughness, degree of weathering, and joint degradation collectively determine successful plant establishment. The survey found many herbaceous species preferring walls with dense crevices and loose surfaces, such as *Youngia japonica* and *Pteris multifida*, commonly occurring on heavily weathered or poorly mortared walls. This reliance on joints reflects selective sensitivity to wall microstructure and demonstrates their niche construction ability under urban disturbance. Retaining walls, which showed the highest diversity, are typically slope-facing, retain higher moisture, and possess stable structures, providing relatively consistent site conditions. In contrast, smooth or highly ornamental facades such as street buildings and fences support fewer plants, indicating that spontaneous plants are not indiscriminately distributed but filtered by habitat structure. From a functional and evolutionary perspective, wall vegetation exhibits clear differentiation among life forms. Herbaceous species are phylogenetically dispersed across multiple lineages, indicating broad ecological tolerance and repeated independent adaptation to vertical wall habitats. In contrast, climbers and woody species show stronger phylogenetic clustering, suggesting that successful establishment on wall surfaces is constrained by conserved traits related to climbing ability, structural support, or rooting strategies. These dominant lineages commonly possess traits such as strong vegetative reproduction, small propagules, short life cycles, or shallow root systems, enabling rapid exploitation of limited space and resources under highly constrained wall microhabitats [42,43]. For example, vines like *Parthenocissus tricuspidata* and *Causonis japonica* quickly spread along walls occupying vertical space; drought-tolerant herbs such as *Corydalis eduli* and *Acalypha australis* complete their life cycles within shallow crevices. Efficient use of spatial and resource niches is key to population establishment in wall microhabitats.

In local greening practices, integrating spontaneous wall plants can complement conventional vertical greening systems. Current urban green walls largely depend on planting modules and irrigation, incurring high maintenance costs and relying on horticultural species, challenging long-term sustainability. Spontaneous wall plants, having undergone natural selection under urban conditions, demonstrate significant local adaptation, strong reproductive ability, and low ecological risk [44]. Promoting their application through selective preservation and seed source guidance can reduce construction material and maintenance costs while enhancing urban greenery’s ecological resilience and regional identity. Moreover, spontaneous plants on walls offer novel support for urban biodiversity conservation [45]. Walls as vertical ecological interfaces connect ground and aerial habitats, potentially serving as movement corridors and refugia for insects, birds, and other fauna [36]. Conserving and managing these plant communities can enhance urban ecological network connectivity without additional land demand. Additionally, spontaneous wall flora exhibits unique landscape values. Many native species display attractive ornamental traits in natural states—*Orychophragmus violaceus* blooms purple flowers in spring, *Corydalis eduli* have purplish double flowers, and *Parthenocissus tricuspidata* shows red foliage in autumn—creating seasonally dynamic vertical landscapes. These plants establish stable populations with minimal maintenance, their natural growth forms contrasting sharply with urban hardscapes, offering dual appeal of ecological beauty and wild charm.

In summary, spontaneous plants not only display excellent ecological adaptability and esthetic qualities but also their local origin, low maintenance, and sensitivity to microhabitats align naturally with current urban ecological development trends. Emerging urban ecological concepts emphasize embedding natural processes and enhancing ecosystem self-organization within urban spaces [46]. As vegetation types form through natural succession, spontaneous plants exemplify this potential. Allowing their natural growth in urban niche spaces aids ecological function restoration and urban resilience while providing a practical pathway toward more localized and sustainable greening systems.

## 4. Materials and Methods

### 4.1. Study Area

The study area is located in Nanjing, the capital of Jiangsu Province, China, situated in the middle and lower reaches of the Yangtze River. It features a typical northern subtropical monsoon climate with four distinct seasons, synchronized rainfall and temperature patterns, an average annual precipitation of approximately 1106.5 mm, an average annual temperature of 16.5 °C, and consistently high relative humidity, all contributing to a marked seasonal climate. The city’s topography is generally elongated from north to south and narrower from east to west, forming a belt-shaped distribution. The landforms consist of alternating low hills and alluvial plains. Soil types vary across regions: yellow-brown soils dominate the northern and central areas, while red soils are more prevalent in the southern part adjacent to Anhui Province. Influenced by the warm and humid climate and diverse terrain, Nanjing is one of the most floristically rich areas in southeastern China. The regional vegetation types include deciduous broad-leaved forests, evergreen broad-leaved forests, and coniferous forests, with deciduous species being predominant [47].

The research site is located within the old urban district of Nanjing’s main city area. Unlike the orderly “planning first, construction material later” layout typical of new urban districts, the old district has evolved organically, featuring a bottom-up spatial pattern with complex textures, fragmented land ownership, and irregular street networks. This spatial configuration has created numerous irregular marginal spaces, including abandoned walls, building facades, retaining walls, and city walls, encompassing a variety of wall types. These walls form important elements of the city’s vertical landscape, providing unique three-dimensional interfaces for the attachment, growth, and dispersal of spontaneous vegetation. Based on preliminary field observations, the wall environment in the old district exhibits multiple overlapping characteristics, including dense historical buildings, diverse wall types, and partially preserved green systems, collectively forming a distinctive urban ecological context. Spontaneous vegetation typically colonizes narrow gaps and crevices along street corners, courtyard edges, hillside slopes, historical relics, and factory fences. Under conditions of limited human management, poor soil quality, and spatial constraints, these plant communities establish relatively stable habitats, forming a significant part of the city’s informal green space system. They are of great importance for studies on urban biodiversity, low-maintenance greening, and vertical greening strategies [48].

### 4.2. Data Collection

#### 4.2.1. Sample Plot and Quadrat Setting

This study investigated the species composition and structural characteristics of spontaneous vegetation on urban walls in the main built-up area of Nanjing. Given the sparse and highly discontinuous distribution of wall plants, conventional systematic-grid or equidistant sampling methods are insufficient to achieve adequate coverage in such contexts [49]. Therefore, a reconnaissance survey was conducted in August 2024. Using a combination of urban land use maps and on-site inspections, we defined the study area, identified key neighborhoods, and established walking transects. During the reconnaissance phase, we also assessed wall accessibility and visibility, aiming to include a diverse range of wall types and land use categories to ensure spatially representative sampling. In the formal survey phase, investigators followed the predefined transects on foot, recording wall vegetation occurrences and associated environmental variables. As vegetation-free walls comprise a large proportion of vertical surfaces, only those hosting spontaneous plants were retained as valid samples to enhance ecological relevance and survey efficiency. Two rounds of vegetation surveys were conducted: the first from September to December 2024, and the second from March to May 2025. The spring survey followed the same routes and protocols as the autumn one, with additional visits and checks to account for seasonal variation in plant assemblages. The specific time and related data for the vegetation survey have been uploaded in the Supplementary File (Table S1).Although summer and winter were not surveyed, the chosen seasons capture the key periods of growth and senescence for most wall plant species in the study region, allowing assessment of primary seasonal dynamics while acknowledging the limitation of incomplete year-round coverage. Samples were collected across wall surfaces at heights ranging from approximately 0.1 m to 2.5 m above the ground, excluding the top and base edges to avoid potential edge effects. At each surveyed site, trees and large shrubs were recorded across the entire wall face, with their abundance and spatial distribution noted, while herbs, vines, and small shrubs were surveyed using standardized quadrats. In addition, the existing vegetation around the wall base and in the adjacent ground areas was recorded to characterize the surrounding habitat and potential seed sources. Following Segal’s recommendation for minimum plot size on vertical walls [50], we established 2–4 rectangular quadrats measuring 4 m × 1 m on each wall, depending on vegetation uniformity and wall dimensions. To minimize edge effects, a 10 cm buffer zone was left unsampled above and below each quadrat. Within each quadrat, we recorded species identity, percent cover, and precise growth position. In total, 321 quadrats were established across 100 walls.

**Figure 7:**
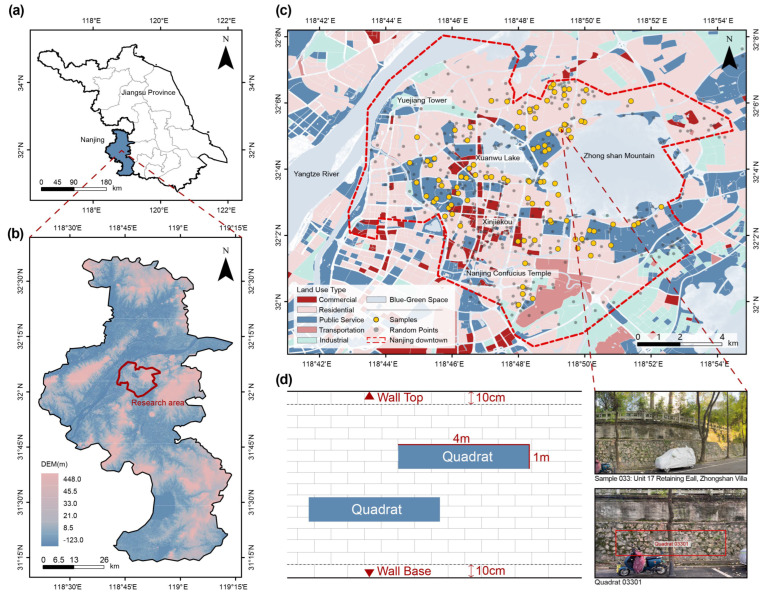
Study area, topographic background, land use, and sampling design. (**a**) Administrative map of Jiangsu Province. (**b**) Elevation map of Nanjing City. (**c**) Land-use types and spatial distribution of wall vegetation sampling sites in the study area. (**d**) Illustration of sampling plot selection and field photo of a representative sample.

To assess the spatial representativeness of our sampling scheme, all quadrat coordinates were imported into a GIS platform. Using ArcGIS Pro 10.8 [51], we generated stratified random points based on urban land use categories and overlaid them with the sampling locations and land use map [52] (Figure 7). The overlay shows that the 100 vegetated walls are distributed across all major land use categories and city districts. Sampling density closely matches the stratified random points, except in high-rise commercial cores—such as the Xinjiekou area, the city’s primary retail and business center—where green infrastructure is notably scarce. Overall, the dataset demonstrates strong spatial representativeness.

#### 4.2.2. Vegetation and Environmental Variables

Plant identification was conducted with reference to Flora of Jiangsu [53], Flora of China [26], and the Catalogue of Naturalized and Invasive Plants in China [54], supplemented by relevant literature and online databases. All recorded species were taxonomically classified by family and genus. During fieldwork, systematic photographic documentation was carried out, capturing general quadrat views, vertical vegetation structure, key morphological traits of herbaceous species, and notable ornamental features. All images were archived by quadrat ID, with filenames indicating the quadrat number and corresponding scientific name.

Environmental variables were recorded at the quadrat level, including geographic coordinates, survey time, and weather conditions (air temperature, relative humidity, and sunlight intensity recorded during fieldwork and cross-checked with data from the Nanjing Meteorological Station). Following protocols used in studies of spontaneous wall vegetation in Chongqing [21], variables were grouped into four categories: intrinsic wall attributes, external wall attributes, joint-related features, and management-related factors. Each variable is defined with its abbreviation, description, and value range in Table 2. Quantitative variables such as wall height were directly measured, while others—such as weathering and human disturbance—were assessed on a five-point Likert scale. Although these assessments involve subjective judgment, all scoring was conducted consistently by the same observer following a standardized protocol, minimizing potential bias.

#### 4.2.3. Statistical Analysis

Plant and environmental data were organized and managed using Microsoft Excel 2021. A phylogenetic tree was constructed based on species names using the V.PhyloMaker2 package [55] and visualized Via the iTOL platform [56]. Mantel tests assessed correlations between plant community groupings and environmental variables, complemented by Pearson’s correlation to explore interrelationships among environmental factors. Variation partitioning was conducted with the varpart function in the vegan package to quantify the relative contributions of four environmental variable groups to species distributions. After selecting the appropriate ordination method through Detrended Correspondence Analysis (DCA), Canonical Correspondence Analysis (CCA) was applied to evaluate environmental influences on community composition. Multiple linear regression models were used to assess the explanatory power of 17 environmental variables on biodiversity indices across seasonal datasets. Community diversity was characterized using Shannon, Simpson, Patrick, and species richness indices. All statistical analyses were performed in R (version 4.4.3).

## 5. Conclusions

In summary, this study highlights the important role of urban walls as components of informal green spaces in maintaining urban biodiversity and providing ecological niches. Observed plant assemblages on Nanjing’s walls exhibit distinct species differentiation and structural characteristics, with inherent wall attributes and micro-scale structures significantly influencing species composition. Notably, wall joints and weathering levels appear to facilitate the establishment of herbaceous plants. Meanwhile, spontaneous plants demonstrate ecological adaptability and landscape potential; their local origin, low maintenance requirements, and sensitivity to seasonal variation underscore their practical value for urban facade greening. These observations align with contemporary urban ecological paradigms emphasizing urban ecology [5], nature-based solutions [6], and urban rewilding [7]. Compared to conventional green walls relying on modular structures and irrigation systems, spontaneous vegetation may better reflects the self-organizing capacity of ecological processes, offering potential for greater sustainability and site-specific adaptability.

Limitations and future research: This study has certain limitations. The long-term stability of spontaneous plant communities lacks continuous dynamic monitoring, and potential impacts on wall structural integrity warrant further assessment. Additionally, spontaneous vegetation remains underrecognized within public management frameworks, with limited societal acceptance and policy support restricting its broader application in urban practice. Future research should focus on succession patterns and functional traits of wall plant communities across diverse urban contexts and wall types, evaluate their ecosystem service contributions, and explore facade greening design frameworks adapted to local conditions. Moreover, advancing management concepts and improving policy mechanisms are essential to integrate wall and crevice habitats into urban ecological networks, enhancing the sustainability and localization of urban greening while safeguarding cultural heritage and ecological security. Furthermore, this study did not include winter data, which may influence seasonal assessments of diversity and species composition. Also, vegetation-free walls were excluded from the analysis, potentially biasing interpretations toward already colonized habitats. Future work should address these gaps to provide a more comprehensive understanding of urban wall plant dynamics.

## Figures and Tables

**Table 1:** DCA Results.

Statistic	Axis 1	Axis 2	Axis 3	Axis 4
Eigenvalues	0.6813	0.4703	0.3551	0.3296
Explained variation (cumulative)	4.19	7.07	9.26	11.27
Gradient length	3.23	5.06	4.34	3.71
Pseudo-canonical correlation	0.4061	0.6267	0.492	0.3416

## Data Availability

The data can be shared and used. Dataset available on request from the authors. The raw data supporting the conclusions of this article will be made available by the authors on request.

## Supplementary Materials

The following supporting information can be downloaded at: https://www.mdpi.com/article/10.3390/plants15040541/s1, Table S1: Vegetation Survey Schedule.
